# Endophytic bacteria isolated from *Urtica* *dioica* L.- preliminary screening for enzyme and polyphenols production

**DOI:** 10.1186/s12934-023-02167-2

**Published:** 2023-08-30

**Authors:** Olga Marchut-Mikołajczyk, Magdalena Chlebicz, Monika Kawecka, Agnieszka Michalak, Filip Prucnal, Maciej Nielipinski, Jakub Filipek, Michalina Jankowska, Zofia Perek, Piotr Drożdżyński, Natalia Rutkowska, Anna Otlewska

**Affiliations:** 1 Biotechnology Students Association Ferment, Faculty of Biotechnology and Food Sciences, Lodz University of Technology, Wolczanska 171/173, 90-530 Lodz, Poland; 2 Institute of Molecular and Industrial Biotechnology, Faculty of Biotechnology and Food Sciences, Lodz University of Technology, Stefanowskiego 2/22, 90-537 Lodz, Poland; 3 Institute of Fermentation Technology And Microbiology, Faculty of Biotechnology and Food Sciences, Lodz University of Technology, Wolczanska 171/173, 90-530 Lodz, Poland

**Keywords:** Bacterial endophytes, *Urtica dioica* L., Metabolites, Polyphenols

## Abstract

Endophytes, especially those isolated from herbal plants, may act as a reservoir of a variety of secondary metabolites exhibiting biological activity. Some endophytes express the ability to produce the same bioactive compounds as their plant hosts, making them a more sustainable industrial supply of these substances. *Urtica dioica* L. (common stinging nettle) is a synanthropic plant that is widely used in herbal medicine due to the diversity of bioactive chemicals it contains, e.g., polyphenols, which demonstrate anti-inflammatory, antioxidant, and anti-cancerous capabilities. This study aimed at isolating endophytic bacteria from stinging nettles for their bioactive compounds. The endophytic isolates were identified by both biochemical and molecular methods (16S rRNA) and investigated for enzymes, biosurfactants, and polyphenols production. Each of the isolated bacterial strains was capable of producing biosurfactants and polyphenols. However, three of the isolated endophytes, identified as two strains of Bacillus cereus and one strain of Bacillus mycoides, possessed the greatest capacity to produce biosurfactants and polyphenols. The derivatized extracts from culture liquid showed the 1.633 mol l^−1^ (9.691 mg l^−1^) concentration of polyphenol compounds. Therefore, the present study signifies that endophytic *B. cereus* and *B. mycoides* isolated from *Urtica dioica* L. could be a potential source of biosurfactants and polyphenols. However, further study is required to understand the mechanism of the process and achieve efficient polyphenol production by endophytic bacteria.

**Supplementary Information:**

The online version contains supplementary material available at 10.1186/s12934-023-02167-2.

## Introduction

Plant tissues include a fascinating group of microbes known as endophytes, which live there without harming the health or functionality of the host plant [1, 2]. Every plant is thought to harbor endophytes, which may also be species-specific [3]. Endophytes are classified into three major groups: obligatory, facultative, and opportunistic [4]. These microorganisms do not necessarily spend their entire life cycle inside plants, but at least a portion of it [4, 5]. The primary entry points for potential endophytic bacteria into the plant include naturally occurring apertures (pores and hydathodes), wounds, micropores, and abiotic mechanical damage (e.g. caused by hail). Most likely, the most significant entry point is through micropores and wounds that have already formed at an early stage of root development [6]. Some endophytic microorganisms can be transmitted e.g. by tubers, bulbs, rhizomes or cuttings, these parts, and thus the offspring, will be inhabited by endophytes. As a result, endophytes can be extracted from all plant tissues [4]. The most typical isolated microbes were bacteria and fungus, but actinomycetes [7, 8], archaea [9] and algae [3] were also found.

Endophytes have lately piqued the interest of the microbial chemistry community due to their enormous potential to aid in the development of novel bioactive chemicals. Their immense potential to contribute to the discovery of new bioactive compounds. This group of microorganisms can function as the biological defense of the plant by producing antibiotics or hydrolytic enzymes to prevent microorganisms, insects, and nematodes from infecting plants. Endophytes can also produce different secondary metabolites, including plant growth promoting agents; and play an important role in atmospheric nitrogen fixation [10, 11].

The incredible diversity of endophytes and their applications is still being studied. However, the industrial potential of endophytic bacteria or fungi is already being used in many industries [12]. For instance, endophytic bacteria can synthesize plant growth promoters and biocontrol agents for use in agriculture [13]. Numerous groups are researching how to use these microbes to create organic molecules, that can be used for biofuels production [14]. Additionally, some research [15, 16] has demonstrated the potential of endophytic enzymes or endophytes themselves as bioremediation agents. However, pharmacology is probably at the forefront of endophyte research, as those microorganisms may be considered a source of new antibiotics, anticancer agents and bioactive compounds [17, 18]. The ability of some endophytes to produce the same bioactive compounds as their plant hosts has also been demonstrated, making them a more sustainable industrial supply of these substances [19–23]. The most well-studied example is paclitaxol, which was first produced by *Taxus* species, but is now known to be synthesized by numerous endophytic fungi connected to those plants [24–26]. Since plants require a lot more resources to develop and flourish than microbes do, it would be helpful to identify and use certain endophytes to produce and optimize the production of various plant-associated chemicals (including medications).

One of the numerous plants widely used in herbal therapy is *Urtica dioica* L. (stinging nettle), which is known to be a host to endophytic microbes, particularly fungus [27–29]. Nettle has been shown to induce diuresis and saluresis [30, 31], as well as to lessen inflammation, lower blood sugar levels, and function as an antihemorrhagic agent [31, 32]. The chemical composition of *U. dioica* L. extracts includes a number of acids (formic, malic, and oxalic), polyphenols (kaempferol, quercetin, caffeic acid, and chlorogenic acid), biogenic amines (acetylcholine, histamine, and serotonin), and numerous other bioactive components [33, 34]. Extracts of *Urtica dioica* L. contain significant amounts of polyphenols. Numerous studies [35, 36] have demonstrated the anti-inflammatory, antioxidant, and anti-cancerous capabilities of these chemicals, which are frequently found in plants, including fruits and vegetables. Although polyphenols are frequently used in medicine, they are also being researched as possible food preservatives [37]. Polyphenols must still be extracted from plants in order to be used in pharmaceutical production, therefore creating novel biotechnological techniques that use microorganisms as producers might assist to lower the cost and environmental effect of such chemicals.

In this study, endophytic bacteria from *Urtica dioica* L. (stinging nettle) were isolated, their biochemical and molecular identification were determined, and the isolates were then analyzed for their ability to produce hydrolytic enzymes, surface active, and polyphenolic compounds.

## Materials and methods

### Plant materials

The healthy *Urtica dioica* L. plants were collected from three different places within the Lodz voivodship: (1) Kopysc (51°36′37.0″N 19°03′15.8″E), (2) Kwiatkowice Las (51°44′32.5″N 19°08′39.8″E), (3) Orpelow (51°38′31.9″N 19°11′56.4″E), in Poland. Whole plants were carefully dug out, ensuring no disruption of tissue continuity, and transported to the laboratory at the Lodz University of Technology. Immediately after, the endophytes isolation was conducted.

### Isolation of endophytic bacteria

Plants were washed under running tap water to remove any residues and soil particles. The plants were separated into stem, leaf and root parts and subjected to surface sterilization. The sterilization procedure was as follows: 70% ethanol (3 min), 6% NaClO (6 min) and 70% ethanol (30 s), fivefold rinsing in sterile distilled water. Within a sterile scalpel, sterilized plant tissues were cut into 1 cm pieces, placed on plates with NB agar medium, and incubated for 6 days at 30 °C. By taking 100 µl of water from the final wash onto NB media and checking for potential microbial growth, the effectiveness of the sterilization procedure was confirmed. Obtained endophytes were selected and streaked to pure strains [15, 38].

### Morphological and biochemical characterization of *Urtica dioica* L. endophytic bacteria

#### The Gram staining

Bacterial biomass was suspended in a drop of distilled water and smeared on skimmed slide glass and air-dried. The slide was heat-fixed by moving through a flame. A small amount of crystal violet was applied to the smear for 2 min. After that time, crystal violet was washed off with distilled water and iodine was added (2′). After two minutes time, samples were gently washed off and 95% ethanol was used to decolorize the sample (30 s). After removing alcohol with distilled water, carbol fuchsin was added [39]. After washing off with distilled water, the samples were observed under a microscope.

#### Biochemical analysis

Using analytical profile index (API) kits, the isolated bacterial strains' ability to use carbon sources and their enzyme activities were examined. According to the manufactures instructions, tests on the API 20E and API 50CHB E profiles were conducted (three times each) at 30 °C for 48 h.

#### Starch hydrolysis

The ability of endophytes to produce amylase was conducted by growing microorganisms on a medium containing as follows: 1% soluble starch, 0.2% yeast extract, 0.5% peptone, 0.05% MgSO_4_, 0.05% NaCl, 0.015% CaCl_2_, and 2% agar (pH 7). Microorganisms were incubated on agar plates at 30 °C for 48 h. Subsequently, plates were flooded with iodine solution for 1 min and then excess was poured off [40].

#### Lipolytic activity

The ability to produce lipase was verified by incubating microorganisms on a basal medium with the addition of 1% of Tween 80 at 30 °C for 7 days [41].

#### Hemolysis activity

Microorganisms were incubated on blood agar plates containing 0.5% peptone, 0.3% yeast extract, 1.5% agar, and 0.5% NaCl (pH = 7.6) with the addition of 5% (v/v) sheep blood. Plates were incubated at 30 °C for 5 days [42].

#### Protease activity

The ability of endophytes to produce extracellular proteases was analyzed by incubating microorganisms on plates with Skim Milk Agar (SMA) medium at 30 °C for 48 h [43].

### Liquid cultures

Selected bacterial endophytes were grown in a liquid LB medium. 40 ml of medium was transferred into fermentation flasks, sterilized (15 min, 121 °C), and inoculated with 0.6 ml of a 24-h pre-culture (OD_600_ 0.8) of the investigated microorganisms. Endophytes were cultured for 24–96 h at 30 °C on a rotary shaker, 180 rpm. Additionally, one set of cultures was supplemented with AlCl_3_, as an indicator of chlorogenic acid [44]. Liquid cultures were analyzed for the ability of isolated bacterial endophytes to produce biosurfactants and polyphenols.

#### Emulsifying activity

The Pearce and Kinsella modified method (Pearce and Kinsella, 1978) was used to determine the emulsifying activity. The reaction mixture contained 3 ml of the culture liquid, 1 ml of diesel oil, and 1 ml of 0.1 M phosphate buffer (pH 7). The mixture was homogenized in a Yellow Line DI 18 Basic homogenizer for 30 s at 18,000 rpm, and then 0.1 ml of the homogenate was added to 1 ml of 0.1% sodium dodecyl sulphate (SDS). On the UV/VIS T80 + spectrophotometer, the OD_500_ against water as a control sample was measured [45, 46].

#### Polyphenols contents

The Folin–Ciocalteu method, as described by Singleton and coworkers [47] was used to determine the total polyphenol content in liquid culture. 60 ml of distilled water H_2_O, 1 ml of culture, and 5 ml of the Folin–Ciocalteu reagent were added to a 100 ml volumetric flask. After a two-minute wait, 15 ml of 20% sodium carbonate was added. The flask was then filled to the 100 ml mark and kept in the dark for two hours. After this time absorbance was measured at λ = 760 nm.

#### Derivatization of obtained products

A post-culture medium was treated with methanol and esterified with trimethyl sulfonium hydroxide (TMSH) in order to evaluate the polyphenolic compounds’ content. After extracting with 100 ml of hexane and separating the organic phase, 10 µl of the extract was mixed with 200 µl of tert-butyl methyl ether and 50 µl of TMSH and heated at 60 °C for 30 min. Then GC/MS analysis was performed [48].

#### GC/MS analysis

The GC/MS analysis was performed using a Thermo Trace GC Ultra/DSQ II chromatograph (Thermo Fisher Scientific, Waltham, MA, USA).

Operating parameters of the gas chromatography were adapted as follows: column—non-polar stationary phase Rxi–1 ms (length 60 m, internal diameter 0.25 mm, film thickness 0.25 μm, Restek Corp., Bellefonte, PA, USA), injector temperature: 280 °C, FID detector temperature: 300 °C, carrier gas—helium 5.0, constant pressure 300 kPa and split ratio 1:50, oven temperature program was 50 °C for 3 min, 50 to 300 °C at 4°/min, 300 °C for 10 min. Mass spectrometry parameters: ion source temperature 200 °C, ionization energy 70 eV. The quantity of the individual components was achieved using a flame-ionization detector connected through the MS-FID splitter (SGE Analytical Science, Ringwood, Melbourne, VIC, Australia). Databases from the NIST Library (RRID:SCR_014680), Wiley 8th edition, and the Adams 4th edition were used. All samples were injected three times.

### Antioxidant and antimicrobial activity of endophytic post-culture supernatant lyophilisates

To evaluate the antioxidant and antimicrobial activity of supernatants, lyophilized, cell-free fresh supernatants were used. The obtained culture supernatants lyophilisates (CSL) were dissolved in 95% methanol to produce solutions with concentrations of 2.5 mg/ml, 5 mg/ml, 10 mg/ml, and 20 mg/ml, which were then sterilized at 121 °C for 15 min. A standard curve was prepared using ascorbic acid, and the ABTS radical scavenging ability was expressed as mg equivalent/g.

#### ABTS radical scavenging activity

Antioxidant activity was determined following a slightly modified method by Saeed et al. (2012). A 7.0 mM stock solution of ABTS^**.**^+ radical cations was prepared and incubated for 16 h at room temperature in the dark. The stock solution was diluted with 50% methanol to obtain an absorbance of 0.70 at λ = 745 nm for testing. Free radical scavenging activity was assessed in a microcuvette by mixing 100 μl of the test sample with 200 μl of ABTS working standard. Simultaneously, a blank test was conducted by substituting the tested solutions with distilled water. After mixing the solution for one minute, the absorbance was measured for the next six minutes. The percentage of inhibition was computed using the following formula: AA (%) = [(A_ABTS+_ – A_x_)/ (A_ABTS+_)] × 100 [49–51].

#### Antimicrobial activity

The antimicrobial activity of CSLs was evaluated using the disk diffusion method. The sterilized CSLs solutions were placed onto 5-mm-diameter sterile filter paper discs. As a base layer, 10 ml of Mueller-Hilton agar medium was placed into sterile Petri dishes, followed by 15 ml of seeded medium containing 10^5^ CFU/ml of medium. On top of Mueller-Hilton agar plates, sterile filter paper discs containing 2.5, 5, 10, or 20 mg/ml of the solutions were deposited. The positive control consisted of filter paper discs laden with 5 g of gentamycin. Plates were stored at 5 °C for two hours to allow plant extracts to diffuse and then incubated at 35 °C for twenty-four hours. The presence of inhibition zones was measured using a Vernier caliper and interpreted as evidence of antibacterial activity [52–54].

### Seed germination test

Germination enhancement of three plants: *Lepidium sativum*, *Allium schoenoprasum*, and *Beta vulgaris* was evaluated according to the PHYTOTOXKIT^®^ methodology [15, 55]. The highest concentrations of CSLs as above (20 mg/ml) were used for seed enhancement germination evaluation (see Additional file 1).

### Molecular characterization

Selected endophytes—strains numbered 2, 5 and 7- were grown in liquid nutrient broth (130 rpm, 30 °C, 24 h). Cultures were collected at OD_600_ 2.531. The DNA was extracted by following the manufacturer’s instructions of the GeneMATRIX Bacterial & Yeast Genomic DNA Purification Kit (EUR_X_®, Molecular Biology Products, Gdansk, Poland). DNA concentration was measured with μDrop™ (Thermo Fisher Scientific, Waltham, MA, USA).

In order to identify microorganisms, PCR was run using primers described in Table 1. The expected amplified products of PCR are shown in Table 2. PCR was performed in C1000 Touch™ Thermal Cycler (BIO-RAD). The conditions for amplification with selected primers were 95 °C for 2 min 15 s, 35 cycles at 94 °C for 75 s, 48 °C for 30 s, 58 °C for 45 s and 72 °C for 75 s, and a final extension step at 72 °C for 5 min. Each PCR was performed in a volume of 50 µl as follows: ~ 0.015 μg template DNA, 25 µl 2 × AmpliTaq Gold® 360 Master Mix (Thermo Fisher Scientific, Waltham, MA, USA), 1 µl of each primer (10 μM) and nuclease free water. The amplicons were separated by gel electrophoresis in 1% agarose gel with ethidium bromide staining. PCR products were sequenced commercially at NEXBIO Sp. z o.o. (Lublin, Poland).

**Table 1:** Characterization of primers used in PCR

Primer	Sequence	Length of the starter [nt]	Tm [°C]
Fn3	5′-CAGGATTAGATACCCTGGTAGTCC-3′	24	57.4
F4	5′-CCGCCTGGGGAGTACG-3′	16	53.6
Fn5	5′-ACTCCTACGGGAGGCAGCAG-3′	20	57.9
Fn6	5′-CCAGCAGCCGCGGTAATAC-3′	19	55.4
Rn1	5′-GGCTACCTTGTTACGACTTC-3′	20	51.8
Rn2	5′-TGACGGGCGGTGTGTACAAG-3′	20	55.9
Rn3	5′-GGCGTGGACTACCAGGGTATC-3′	21	58.3

**Table 2:** Expected products of used primers

Primer’s pairs	Expected product’s length [bp]
Fn3 + Rn1	~ 721/743
Fn3 + Rn2	~ 623/636
F4 + Rn1	~ 633/639
F4 + Rn2	~ 532/536
Fn5 + Rn1	~ 1142/1183
Fn5 + Rn2	~ 1073
Fn5 + Rn3	~ 465/473
Fn6 + Rn3	~ 287/298
Fn6 + Rn1	~ 995/991
Fn6 + Rn2	~ 893

### Statistical analysis

For data analysis the Statistica (RRID:SCR_014213) was used. Data were represented as the mean ± standard deviation (SD) of the triplicate samples. For the polyphenol production, the significance of differences between means was evaluated by one-way ANOVA, and post hoc Tuckey test.

## Results

### Isolation and selection of bacterial endophytes

**Fig. 1:**
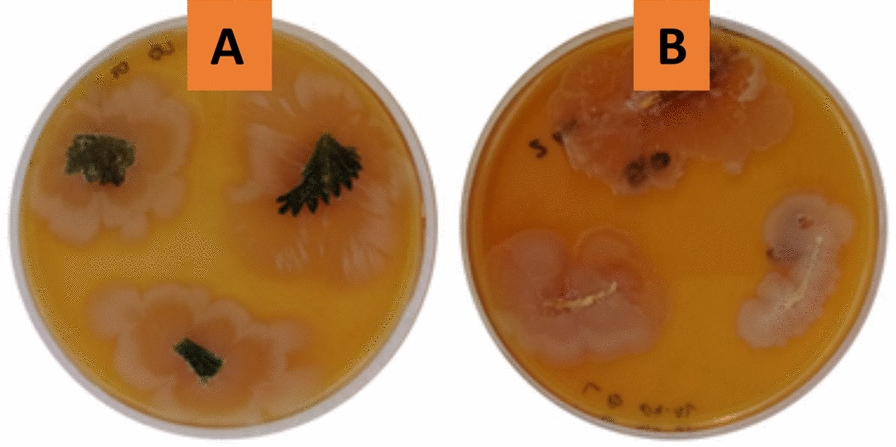
Examples of obtained endophytic microorganisms isolated from various tissues of *Urtica dioica* L.: **A** leaf; **B** root

**Fig. 2:**
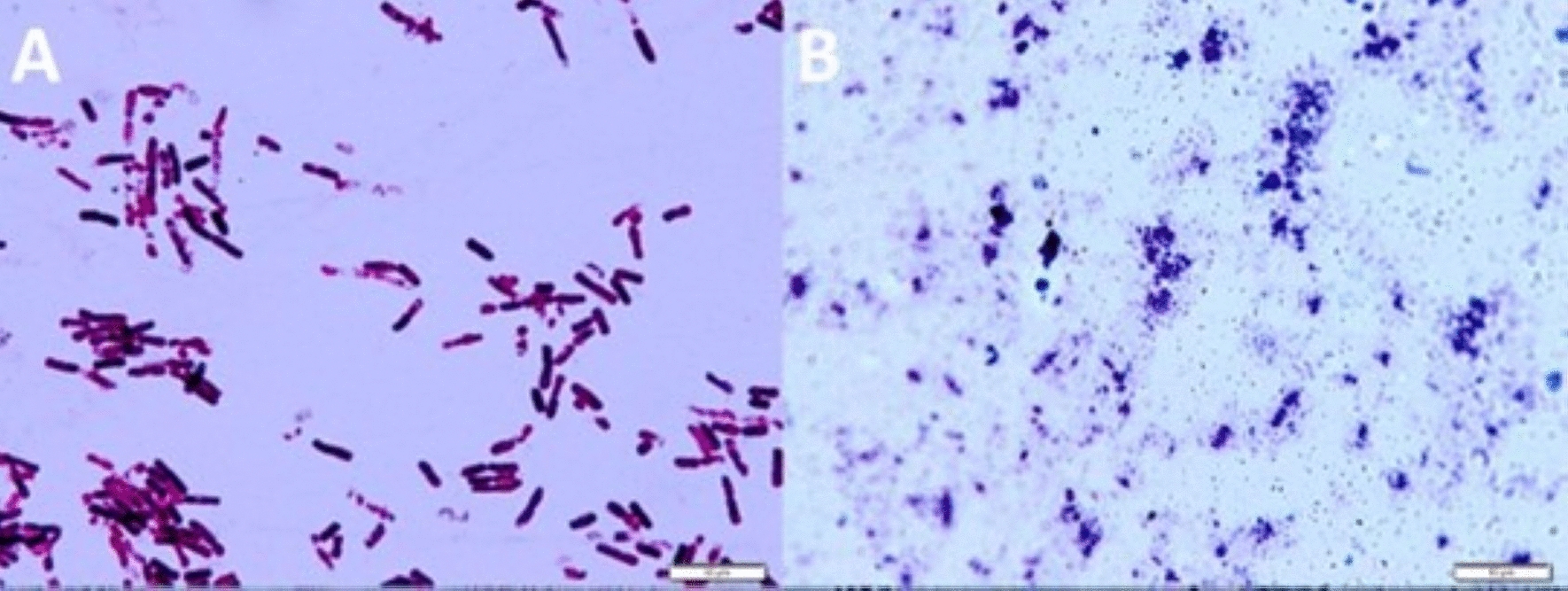
Microscope images of isolated endophytes after Gram staining; **A** org. “2”; **B** org. “3”; scale bar 10 µ

The surface-sterilized fragments of leaves, stems, and roots were placed on petri dishes filled with NB media. Following the incubation time, 20 isolates were collected, of which 7 were bacteria that were used for additional analysis. The rest of the isolates were fungi. Three of bacterial isolates used in further experiments originated from plant dug up in (1) Kopysc, two of them from (2) Kwiatkowice Las and two from (3) Orpelow (Table 3). Figure 1 shows sample petri dishes with the isolated organisms. Gram staining and microscopic examination revealed that both Gram-positive and Gram-negative bacteria were present among isolates (Fig. 2). In order to evaluate the ability of the obtained bacterial endophytes' to produce biosurfactants and polyphenols, 96-h liquid cultures of the endophytes in LB medium were conducted. The 96-h liquid culture experiment showed that every isolate has the potential for biosurfactants production. For the strains marked with numbers “2”, “5”, and “7”, the highest levels of emulsifying activity were obtained after 96 h of cultivation. The highest phenolic compound concentration values were detected for the same strains.

**Table 3:** Characteristics of isolated bacterial endophytes and their liquid cultures

Endophyte No	Plant No	Tissue	Group	24 h		48 h		96 h				
				pH	C_P_ [mol/L]	pH	C_P_ [mol/L]	Without AlCl_3_			With AlCl_3_	
								pH	C_P_ [mol/L]	EA OD500	pH	C_P_ [mol/L]
**2**	**2**	**L**	**G+**	8.5 ± 0.1^a^	**0.325 ± 0.01**^**a**^	8.76 ± 0.1^a^	**1.107 ± 0.09**^**a**^	9.29 ± 0.1^ab^	**0.903 ± 0.02**^**a**^	2.079 ± 0.12	9.28 ± 0.1^ab^	**1.028 ± 0.11**^**a**^
3	2	R	G–	8.36 ± 0.1^b^	0.211 ± 0.06^**a**^	8.42 ± 0.1^b^	0.197 ± 0.06^**a**^	9.24 ± 0.1^ab^	0.456 ± 0.02^**a**^	1.808 ± 0.07	9.24 ± 0.1^ab^	0.499 ± 0.04^**a**^
4	1	R	G–	8.05 ± 0.1^a^	0.193 ± 0.07^**a**^	8.16 ± 0.1^a^	0.205 ± 0.07^**a**^	9.19 ± 0.1^a^	0.408 ± 0.03^**a**^	0.947 ± 0.07	9.19 ± 0.1^ab^	0.477 ± 0.07^**a**^
**5**	**1**	**L**	**G+**	8.42 ± 0.1^a^	**0.650 ± 0.07**^**a**^	8.73 ± 0.1^a^	**0.686 ± 0.07**^**a**^	9.16 ± 0.1^ab^	**0.715 ± 0.09**^**a**^	1.912 ± 0.07	9.17 ± 0.1^ab^	**0.916 ± 0.10**^**a**^
6	1	R	G–	8.31 ± 0.1^a^	0.450 ± 0.07^**a**^	8.54 ± 0.1^a^	0.511 ± 0.07^**a**^	9.12 ± 0.1^ab^	0.297 ± 0.07^**a**^	1.715 ± 0.06	9.16 ± 0.1^ab^	0.308 ± 0.01^**a**^
**7**	**3**	**S**	**G+**	8.12 ± 0.1^a^	**1.633 ± 0.07**^**a**^	8.59 ± 0.1^a^	**0.489 ± 0.07**^**a**^	9.01 ± 0.1^a^	**0.698 ± 0.09**^**a**^	1.996 ± 0.04	9.3 ± 0.1^a^	**0.775 ± 0.10**^**a**^
8	3	R	G+	8.09 ± 0.1^b^	0.187 ± 0.07^**a**^	8.19 ± 0.1^b^	0.107 ± 0.05^**a**^	9.28 ± 0.1^ab^	0.475 ± 0.09^**a**^	1.587 ± 0.10	9.28 ± 0.1^ab^	0.499 ± 0.039^**a**^

L, leaf; R, root; S, stem; G+, Gram positive; G–, Gram negative; C_P,_ Polyphenol concentration [mol/L]; EA, Emulsifying activity

Statistical significant; ab- statistical significant relative to a; insignificant relative to b

Statistically insignificant

It should be noted that the culture's concentration of polyphenolic compounds increased slightly after the addition of aluminum chloride, which may be connected to the formation of more chlorogenic acid (Table 3). Additionally, it is important to note that the pH of the culture fluid increases over the course of the culture, which may be connected to the production of surfactants. Based on the results, three bacterial endophytes—numbered “2”, “5” and “7”—were selected for further analysis.

**Fig. 3:**
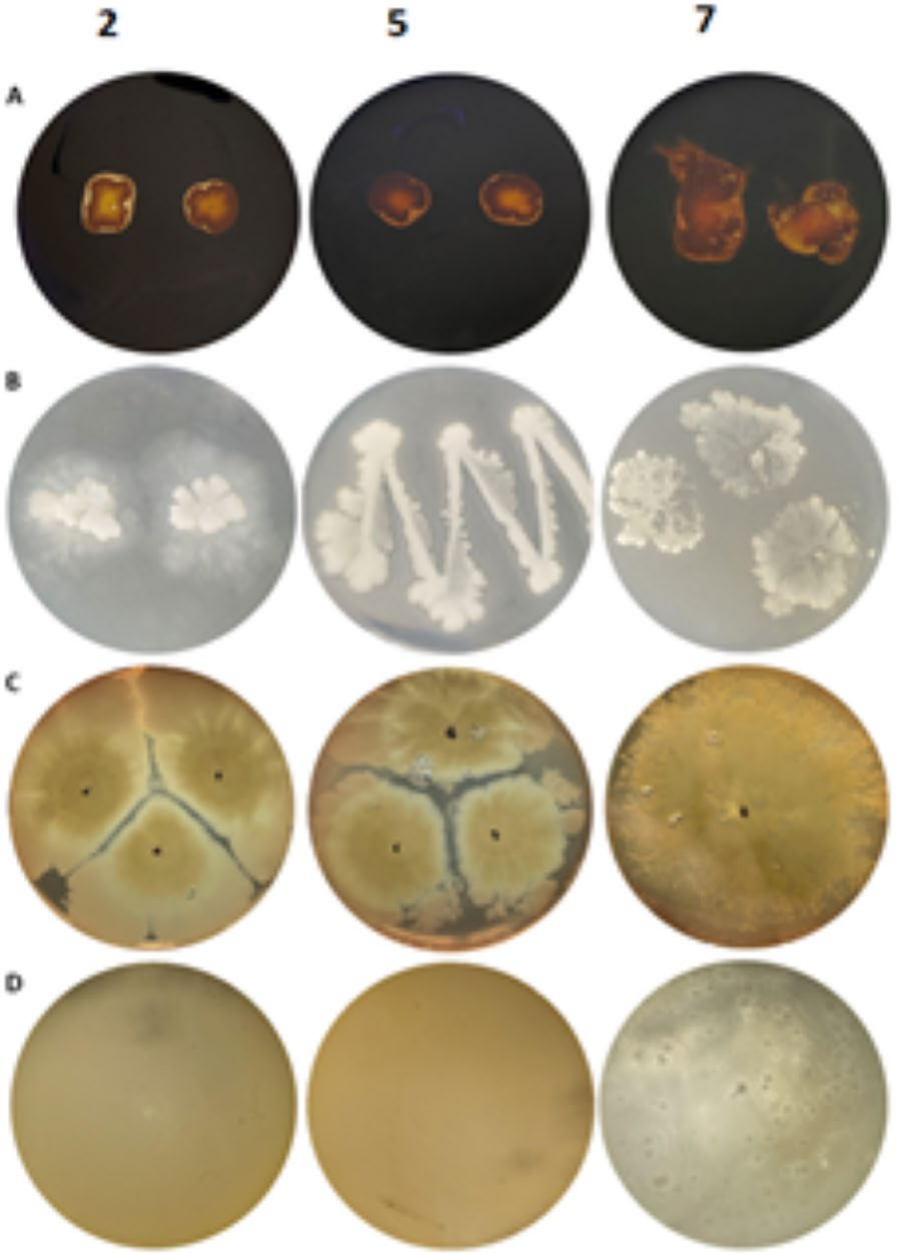
The ability of isolated bacterial endophytes “2”, “5” and, “7” to produce hydrolytic enzymes. **A** amylolytic activity, **B** lipolytic activity, **C** hemolytic activity, **D** proteolytic activity

The ability of the tested bacterial endophytes isolated from *Urtica dioica* to produce selected hydrolytic enzymes—amylolytic, lipolytic, proteolytic, and hemolytic—was assessed in plate cultures on media containing appropriate substrates. All the bacteria exhibited amylolytic and lipolytic activity but no proteolytic activity. Additionally, all the examined strains demonstrate β-hemolytic activity (Fig. 3).

### Antioxidant activity of lyophilized post-culture supernatants of selected endophytic bacteria

Antioxidant activity monitored as ABTS radical scavenging activity showed that CSL from the "2" strain presented the highest antioxidant activity (Table. 4). The maximum ABTS radical scavenging activity 1,936 mg AAE/g was found for a 20 mg/ml methanol solution of CSL from “2” endophyte. The antioxidant activity of the tested solutions was also obtained for the strains denoted by the symbols “5” and “7”, although it was much lower than it was for CSLs of the “2” strain. For methanol solutions of CSLs at a concentration of 2.5 mg/ml, no antioxidant activity was detected for any of the examined endophytic isolates.

**Table 4:** Antioxidant Activity of CSL’s of selected endophytic bacteria by ABTS radical scavenging assay

Endophytic bacteria	CSL solution [mg/ml]	ABTS radical scavenging activity [%]	ABTS radical scavenging activity [mg AAE/g]
“2”	2.5	0	0
	5	6.76 ± 0.31	0.273 ± 0.08
	10	36.28 ± 1.78	1.457 ± 0.01
	20	48.21 ± 1.42	1.936 ± 0.03
“5”	2.5	0	0
	5	4.07 ±	0.165 ± 0.009
	10	21.81 ±	0.886 ± 0.01
	20	29.17 ± 1.84	1.188 ± 0.02
“7”	2.5	0	0
	5	2.69 ± 0.14	0.093 ± 0.008
	10	15.42 ± 0.77	0.591 ± 0.02
	20	19.48 ± 0.69	0.716 ± 0.02

*AAE* ascorbic acid equivalent

### Antimicrobial activity of lyophilized post-culture supernatants of selected endophytic bacteria

**Fig. 4:**
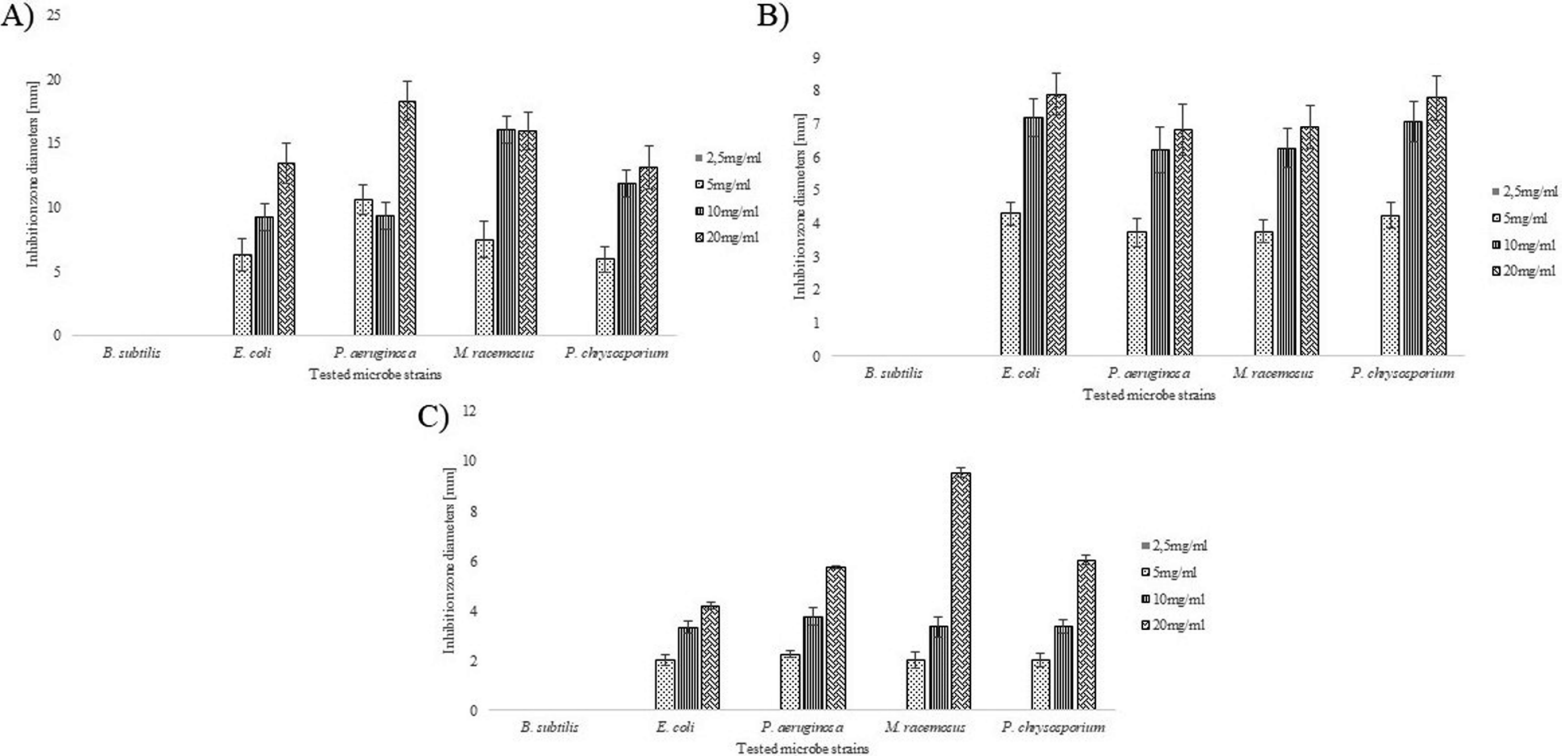
Antimicrobial activity of lyophilized post-culture supernatant of **A** “2” strain **B** “5” **C** “7”

Figure 4 depicts the antimicrobial activity of CSLs derived after cultivating endophytic strains of bacteria labeled “2”, “5”, and “7” using the disc diffusion method. The tested solutions were found to be effective against Gram-negative bacteria, represented by *Pseudomonas aeruginosa* and *Escherichia coli*, and filamentous fungi, represented by *Mucor racemosus* and *Phanerochaete chrysosporium*. The tested CSL’s had no effect on the Gram-positive strain represented by *Bacillus subtilis*.

### Enhancement of seed germination

**Fig. 5:**
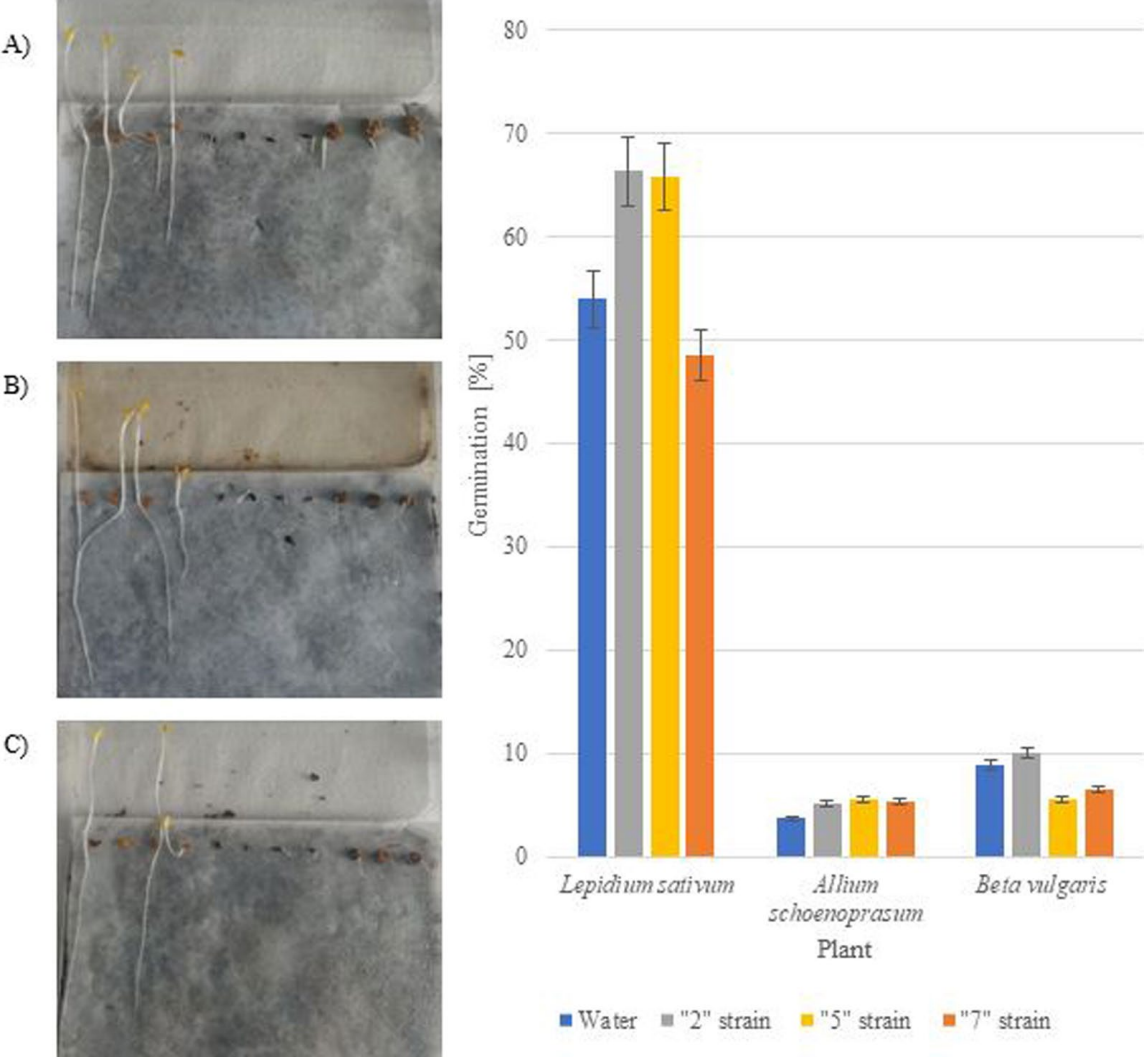
Evaluation of the CSLs impact on germination of *Lepidium* *sativum*, *Allium* *schoenoprasum*, and *Beta* *vulgaris* enhancement

Figure 5 depicts the effect of three CSLs derived from the culturing strains “2”, “5”, and “7” on the germination of seeds of plants indicated in point 2.6 of the Materials and Methods section in comparison to the germination of these plants irrigated with water. Two of the three CLSs (“2” and “5”) examined exhibited better germination-promoting capacity than water. CSL derived from strain “2” had the largest effect on *Lepidium sativum*, increasing it by 13 percent points; a little smaller increase was observed with CSL “5”, which increased by 12 percent points. CSL “7” was shown to have no influence on the germination of the plant. Additionally, the other plants grew to a greater amount in respect to the water; nonetheless, this rise was similar for all CSLs.

### Identification of selected endophytic bacteria isolated from *Urtica dioica* L.

#### Biochemical characterization

Selected endophytic bacteria were identified in two stages. The ability of isolates to produce spores led to the first classification of these bacteria as members of the *Bacillus* family. The first step of the process involved biochemical identifications with the use of a flowchart for identifying Gram-positive bacteria. After that, tests for biochemical parameters and API were conducted (Tables 5 and 6, respectively).

**Table 5:** Biochemical characterization of selected endophytic bacteria isolated from *Urtica dioica* L according to the flowchart for identification of Gram-positive bacteria [56]

No. Endophyte	Amylase	VP-test	Lipase test	Hemolysis	Protease test	Catalase test
2	+	+	+	β -Hemolysis	–	+
5	+	+	+	β- Hemolysis	–	+
7	+	+	+	β- Hemolysis	–	+

**Table 6:** API 20E test results for “2”, “5” and “7” microorganisms

Tests	Active ingredients	Strain No. 2	Strain No. 5	Strain No.7
API 20 E				
ONPG	Beta-galactosidase	–	–	–
ADH	Arginine-dihydrolase	–	–	+
LDC	Lysine decarboxylase	–	–	–
ODC	Ornithine decarboxylase	–	–	–
CIT	Citrate	–	–	–
H2S	Sodium thiosulfate	–	–	
URE	Urease	–	–	–
TDA	Tryptophan deaminase	–	–	–
IND	Indole	–	–	–
VP	Acetoin	–	–	
GEL	Gelatinase	+	+	+
NIT	Potassium nitrate	–	–	

The first biochemical test, starch hydrolysis, gave positive results for all three endophytes. Subsequently, the Voges–Proskauer test was performed. All the samples tested positive for the production of acetyl methyl carbinol, which was consistent with the specific group of strains. As the *Bacillus cereus* group contains biohazard strains, hemolysis activity was performed. The obtained results revealed hemolysis in the organisms "2”, “5" and “7”. The catalase test showed that all the organisms produced catalase. Additionally, API tests were conducted. These biochemical tests enabled the final characterization of the microorganisms. According to the obtained results, the “2” and “7” microorganisms were classified as *Bacillus cereus*.

#### Genotyping

**Fig. 6:**
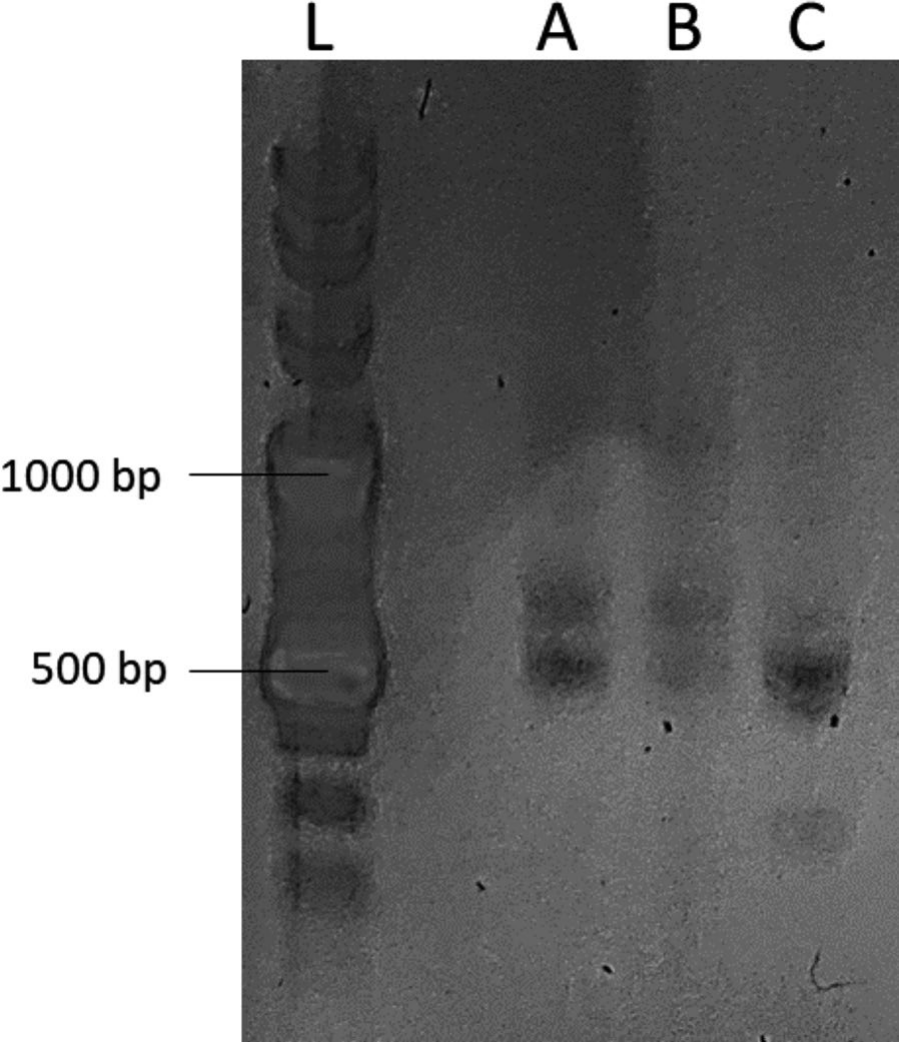
Agarose gel electrophoresis of **A** org. “2”, **B** org. “5” and **C** org. “7”

Gel electrophoresis (Fig. 6) indicated the presence of endophytes of interest with predicted band sizes (Table 2). Amplified products of 16S rRNA’s were sequenced. The obtained sequences were analyzed for homology with the Basic Local Alignment Search Tool and its database. Organism “7” was identified as *Bacillus mycoides*. Strains “2” and “5” were identified as *Bacillus cereus* with an E-value of 100% (GenBank: OP777496) and 99.93% (GenBank: OP777497) percent identity, respectively (RRID:SCR_002760).

Results of genotyping and biochemical tests allowed to identify selected strains as two *Bacillus cereus* strains and *Bacillus mycoides* strain.

### Identification of endophytic bacteria products by gas chromatography coupled with mass spectrometry

Gas chromatography coupled with mass spectrometry revealed that in the two of the three strains post-culture fluids contain polyphenol compounds, namely caffeic acid and chlorogenic acid for strain “2” (retention times of 19.64; 21.71 and 58.55, respectively) and for strain “5” (retention times of 20.00; 21.68 and 58.81, respectively); some of which can be found in nettle extract. Moreover, the post-culture liquid contained fatty acids esters (mainly hexadecenoic, heptadecanoic, and octadecanoic acids) that may be produced by the strains as a product.

**Fig. 7:**
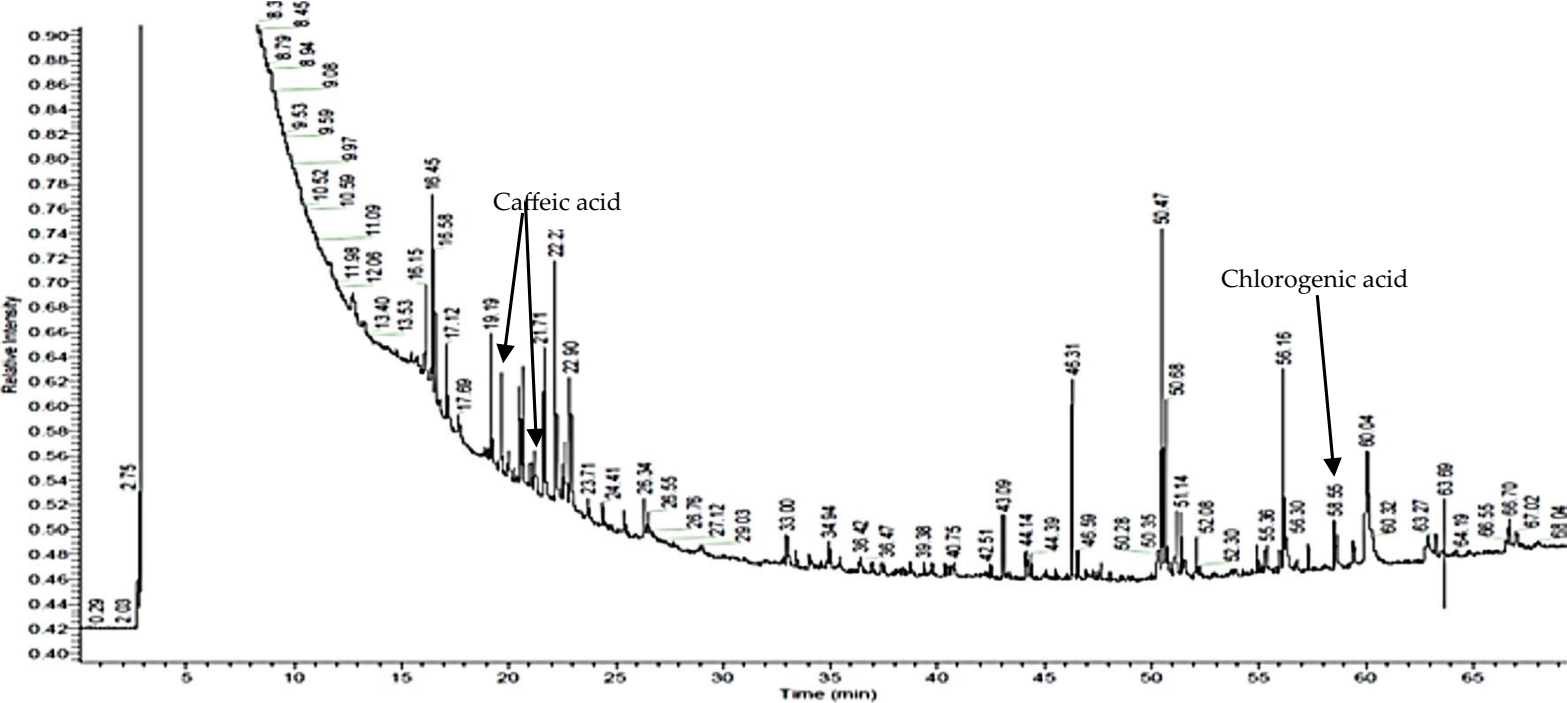
Chromatogram of “2” strain’s post-culture liquid

**Fig. 8:**
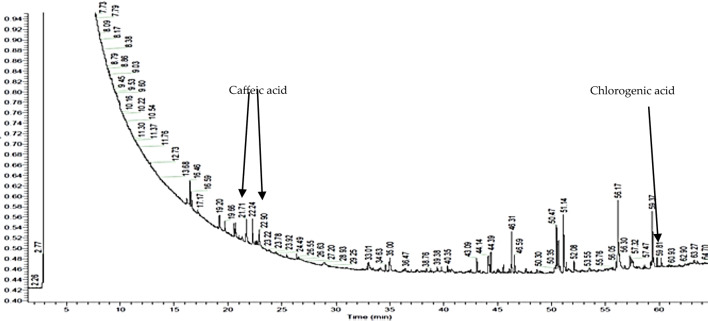
Chromatogram of “5” strain’s post-culture liquid

The analysis thus supports the hypothesis that endophytes are capable of producing compounds similar to or the same as those of the plant host. These results are presented in Figs. 7 and 8 and Tables 7 and 8.

**Table 7:** Compounds identified by GC/MS, present in the post-culture liquid of “2” strain

Compound	Total %	Molecular weight	Retention time [min]	Chemical formula
Caffeic acid	3.38	180.16	19.64	C_9_H_8_O_4_
Hexadecanoic acid, phenylmethyl ester	1.29	170.13	20.68	C_10_H_18_O_2_
Caffeic acid, methyl ester trans	4.95	194.18	21.68	C_10_H_10_O_4_
Hexadecanoic acid, methyl ester	0.79	270.26	46.31	C_17_H_34_O_2_
9,12-Octadecadienoic acid (Z,Z)-, methyl ester	1.3	294.26	50.47	C_19_H_34_O_2_
9-Octadecenoic acid (Z)-, methyl ester	2.02	296.27	50.68	C_19_H_36_O_2_
Heptadecanoic acid, 9-methyl-, methyl ester	6.68	298.51	51.14	C_19_H_38_O_2_
9-Octadecenamide	11.31	281.27	56.16	C_18_H_35_NO
Chlorogenic acid methyl ester	4.29	354.31	58.55	C_17_H_20_O_9_

**Table 8:** Compounds identified by GC/MS, present in the post-culture liquid of “5” strain

Compound	Total Area [%]	Molecular weight	Retention time [min]	Chemical formula
Caffeic acid methyl ester, cis	0.67	180.16	20.00	C_9_H_8_O_4_
Hexadecanoic acid, phenylmethyl ester	0.79	170.13	20.68	C_10_H_10_O_4_
Caffeic acid methyl ester trans	1.95	194.18	21.71	C_17_H_34_O_2_
Hexadecanoic acid, methyl ester	0.59	270.26	46.31	C_19_H_34_O_2_
9,12-Octadecadienoic acid (Z,Z)-, methyl ester	1.1	294.26	50.47	C_19_H_36_O_2_
9,12,15-Octadecatrienoic acid, methyl ester,(Z,Z,Z)-	0.13	296.27	50.68	C_19_H_38_O_2_
9-Octadecenoic acid (Z)-, methyl ester	1.07	298.51	51.14	C_18_H_35_NO
Heptadecanoic acid, 9-methyl-, methyl ester	3.79	281.27	56.16	C_19_H_38_O_2_
Chlorogenic acid methyl ester	0.29	354.31	58.81	C_17_H_20_O_9_

According to the GC/MS results, the strain labeled with “2” was able to produce caffeic acid and chlorogenic acid (Fig. 7, Table 7). Strains labeled with “5” and “7” also were able to produce these compounds, however, the amounts were significantly lower (Fig. 8, Table 8).

Despite the fact that the biosynthesis efficiency of these compounds is rather low and their total share in the chromatogram is less than 8% for caffeic acid and less than 2% for chlorogenic acid, their production by microorganisms can be a very valuable achievement. Thanks to this, the production of polyphenols in a biotechnological way may turn out to be more cost and time effective than extraction from plants. Moreover, the optimization of the process of obtaining these compounds may contribute to achieving even higher yields of polyphenol compounds. The obtained results suggest the possibility that endophytic microorganisms might synthesize compounds that are present in plant extracts.

## Discussion

Endophytic microorganisms can be crucial determinants of plant survival in harsh natural habitats and can be partly (or wholly) responsible for bioactive compounds occurring in plants. Since nettle has been known for years for its beneficial health effects associated with its high polyphenolic content, it is promising to study its endophytes for in vitro synthesis of similar compounds for its more sustainable production on a larger scale. This research is aimed to isolate endophytic bacteria living in *Urtica dioica* L. (stinging nettle), and characterize and identify species showing biotechnological potential.

*Urtica dioica* L. has been recognized as a host plant for various endophytic microorganisms. Zoulikha et al. (2016) isolated bacterial endophytes from *U. dioica*, in which every species identified according to BLAST analyses of its 16S rDNA sequences was a representative of *Bacillus* genus (*B. toyonensis*/*B. thuringiensis*, *B. amyloliquefaciens*, *B. cereus*, *B. pumilus*, *B. methylotrophycus*) [28]. The researchers tested them for their capacity to improve tomato plant development and activity against pathogenic *Agrobacterium* and *Pectobacterium* spp. strains, with *Bacillus methylotrophycus* appearing as one of the potentially beneficial strains [28]. In addition, Naoufal et al. [29] isolated 54 bacterial endophytic strains from *Urtica dioica* L. and selected three having the best antagonistic effect on the phytopathogenic fungus. All selected strains were characterized biochemically and physiologically and assigned to the genus *Bacillus* spp. Analyzed isolates possessed the ability to produce oxidases, catalases, ureases, cellulases and amylases. Moreover, Naoufal et al. [57] isolated 54 endophytes from *Urtica dioica* L. and identified one as *Paenibacillus polymyxa* (also known as *Bacillus polymyxa*), which exhibited great potential with use as biological control agent against *Fusarium wilt*. Viktorova et al. [58] isolated plethora of endophytic bacteria from *Urtica dioica* L. cultivated in different contaminated soil, with the representatives of *Bacillus* genus among them (*B. pumilus*, *B. shackletonii*, *B. cereus*, *B. megaterium*, *B. mycoides*, *B. simplex*, *B. thuringiensis*, *B. weihenstephanensis*), and pointed out similar bioremediation activities between the plant and some of its bacterial endophytes. *Bacillus shackletonii* has shown the highest promise in terms of plant growth promotion properties. Also, Toubal et al. [59] isolated and identified eleven species of bacterial endophytes inhabiting sting nettle. Among them, four species belong to the *Bacillus* genus: *B. anthracis*, *B. megaterium, B. pumilus*—ME, and *B. cereus.* These species were identified by the use of MALDI-TOF/MS. These findings correlate with our research, which established three *Bacillus* strains—one *Bacillus mycoides* and two *Bacillus cereus*—as nettle’s endophytes isolated from leaves and stem according to both biochemical characterization and 16S rDNA sequencing.

However, none of the studies on stinging nettle’s bacterial endophytes concentrated on polyphenols or biosurfactants production.

Biosurfactants are biodegradable and non-toxic surface-active compounds produced by microbes, with applications in environmental protection and agriculture. This class of chemicals can be produced by a wide range of microorganisms, including endophytes. Reports on biosurfactants produced by endophytic bacteria from the *Bacillus* genus primarily address the species *Bacillus subtilis* [60–63]. Serrano and coworkers (2021) demonstrated the ability of endophytic bacteria from the *Bacillus* genus isolated from cocoa trees for biosurfactant biosynthesis, which were identified as *Bacillus velezensis, B. amyloliquefaciens* and *B. subtilis* groups [64]. Marchut-Mikolajczyk and coworkers (2020) described the ability of endophytic *Bacillus cereus* EN18, isolated from *Chelidonium majus*, for biosurfactant production during slop oil biodegradation [65]. However, none of the reports investigated endophytic strains that belonged to the *Bacillus mycoides* genera as potential biosurfactant producers.

Bacteria are not commonly studied for the biosynthesis of polyphenolic compounds since they are not considered natural producers of these compounds and require the use of heterologous pathways [66]. Polyphenols (phenolic acids, flavonoids, stilbenes, lignans) are known to inhibit the growth of bacteria and other microbes by damaging cell walls and shifting metabolic pathways, resulting in cell death. This is the primary issue limiting the high efficiency of microbial production of these chemicals, and as a result, scientific papers on the subject are scarce [67]. Our results show that Gram-positive endophytic bacteria from *Bacillus* spp. are capable of synthesizing in vitro phenolic compounds in the range of 0.325–1.633 mol/dm^3^. *Bacillus cereus* 2KN produces a threefold higher amount of polyphenols in 48 h culture (1.107 mol/dm^3^) than it does 24 h (0.325 mol/dm^3^), however, polyphenols levels do not increase significantly in the 48 h culture of *Bacillus cereus* 5KN. On the contrary, *Bacillus mycoides* produces the highest amount of polyphenols from all tested isolates in 24 h culture (1.633 mol/dm^3^) and that amount drops drastically with the extension of cultivation time to 48 h (0.489 mol/dm^3^). That finding shows the strain’s potential to be the subject of further optimization of polyphenol production. GC/MS analysis revealed that, among the total polyphenol content and some lipid compounds, there is caffeic acid and chlorogenic acid. Production of caffeic acid by genetically modified *E. coli* was investigated by Lin and Yan [68]. In the first step of production using modified *E. coli* strain, they achieve 12.1 mg/dm^3^. Further modifications of this strain allowed for the extraction 50.2 mg/dm^3^ after 2-days cultivation [69]. Another strain used for polyphenols production was *Corynebacterium glutamicum* by Tharmasothirajan and coworkers (2021). With the use of that strain and the application of environmental engineering, they obtained 1.71 g/dm^3^ of resveratrol while biosynthesis in the bioreactor [68].

Some other researchers also managed to assess the polyphenolic potential of endophytic *Bacillus* spp. Two *Bacillus* strains (*Bacillus siamensis*, *Bacillus aryabhatii*) isolated from the *Hoya multiflora* Blume plant showed high phenolic contents of 26.17 ± 0.48 and 16.86 ± 0.59 mg/g of gallic acid equivalents (GAE), respectively. It correlates with the high antioxidant activities of bacterial crude extract [70]. Another endophytic *Bacillus* sp. from *Carica papaya* L. produces not only 69 mg/g of GAE but also shows an antibacterial effect [71]. Nongkhlaw et al. [72] evaluated both epiphytes and endophytes linked with several medicinal plants, the majority of which were *Bacillus* genus members. The amount of total phenolics in aqueous extracts of post-culture liquids of isolated *Bacillus* sp. endophytes is in the range of 10.5–16.0 mg/g of GAE. Rahman et al. (2017) also discovered that *Bacillus subtilis* isolated from *Fagonia indica* generates 243 g/mg of GAE and has significant antibacterial activity. Our findings align with those of these other studies. Particularly the CSL obtained from endophyte “2” demonstrated antimicrobial effects against Gram-negative bacteria and filamentous fungi, which clearly indicates that obtained CSLs have potential antimicrobial activity [73].

Other researchers were able to evaluate the antioxidant capacity of cell-free supernatants as well as we did in our research. Zhou et al. [74] tested cell-free extract of *Lactobacillus plantarum* GXL94 which exhibited significant capture of ABTS radical activity, with ratio of 89.61%. What is more, they evaluated the post-culture supernatant which has 94.63% of ABTS radical scavenging activity. Kim et al. [75] tested fifteen cell free supernatants of lactic acid bacteria which exhibited ABTS radical scavenging activity ranging from 19.69 to 86.26%. Above mentioned activity of only three of the tested strains was lower than 30%. It correlates with our results – in the highest used concentration of CSL (20 mg/ml) the highest value of ABTS radical scavenging activity was 48% for obtained from “2” endophytic bacteria; others were significantly lower—29% and 19% for “5” and “7” endophytic CSL, respectively.

Three endophytic strains of phenol-producing fungi were studied by Garca Latorre et al. (2023). Their studies and other articles demonstrate that polyphenols influence plant growth primarily via environmental stress, defense, and protection against a variety of pests and diseases. Since the experimental conditions were extremely conducive to plant growth and the plants were not subjected to pests and diseases, the protective function of polyphenols may not have been the primary mechanism in this instance. However, the growth of *Lepidium* *sativum* was increased by two of three CSLs—“2” by 13 percent points and “5” by 12 percent points, respectively. It is possible that polyphenols stimulate plant growth in different ways [76, 77].

Our findings also reveal that all three isolates are capable of generating amylases and lipases, which are industrially significant hydrolytic enzymes, which is consistent with previous reports on *Bacillus* sp. as endophytes [78–80]. *Bacillus cereus* is well-known for its enzymatic activity [81, 82], plant growth-promoting traits [83, 84], oil biodegradation [65], as well as for being foodborne pathogen [85]. However, none of the studies demonstrated the potential of endophytic *Bacillus cereus* for polyphenols production. For the isolated *Bacillus cereus* endophytic strains the highest polyphenol concentration was obtained after 48 h for *B. cereus* “2”—1.107 ± 0.087 mol/dm^3^ and after 96 h for *B. cereus* “5”—0.916 ± 0.095 mol/dm^3^.

The genome sequencing of *Bacillus mycoides* isolated from *Lolium perenne* revealed multiple gene clusters involved in displaying plant growth and health, it may have significant potential [86]. Among the polyphenol extracts obtained from bacterial cultures, *Bacillus mycoides* “7” produced the highest concentration of these compounds—1.633 mol/dm^3^ (9.691 mg/dm^3^ GAE) of polyphenols in 24 h of culture (Table 3). Following that, the quantity decreases in 48 h (0.489 mol/dm^3^) and 96 h (0.675 mol/dm^3^) of culture (Table 3).

## Conclusions

With the increasing demand for bioactive compounds and the limited synthesis of plant-active chemicals due to the metabolic and environmental needs of plants, as well as seasonality, it may be essential to produce such compounds using biotechnological methods. The objective of the research was to isolate, characterize, and identify the bacterial endophytes living in *Urtica dioica* L. tissues and evaluate their potential for bioactive compound production (surfactants, polyphenols, and certain hydrolytic enzymes). All seven isolated bacterial endophytes had the potential to produce biosurfactants, but only three showed the ability to produce quite high concentrations of polyphenols. The strains were identified as two *Bacillus cereus* strains and one *Bacillus mycoides*. Further studies are needed to understand the mechanism of polyphenol biosynthesis and their role in plant-endophyte relations to achieve efficient polyphenol production by endophytic bacteria isolated from *Urtica dioica*.

### Supplementary Information

**Additional file 1: Table S1.** API 20 E test results of “2”, “5”, “7” microorganisms.

## Data Availability

The datasets generated during the current study are available in the National Library of Medicine, National Center for Biotechnology Information, https://www.ncbi.nlm.nih.gov/nuccore/OP777496 and https://www.ncbi.nlm.nih.gov/nuccore/OP777497.

## Declarations

### Data collected from plants

No local, national, or international guidelines and legislation procedures were required for the study.
