# Supplementary material for: Endophytic bacteria isolated from Urtica dioica L.- preliminary screening for enzyme and polyphenols production
Source: Microb Cell Fact. 2023 Aug 30;22:169. doi: 10.1186/s12934-023-02167-2 (PMC10466763; doi:10.1186/s12934-023-02167-2)
Supplement: Supplementary file 1 — Additional file 1: Table S1. API 20 E test results of “2”, “5”, “7” microorganisms. [file 12934_2023_2167_MOESM1_ESM.pdf]

Table S1 API 20 E test results of ”2”, “5”, “7” microorganisms

API test	Tests	Active ingredients	Strain No. 2	Strain No. 5	Strain No.7
API 50 CHB V4.1	GLY	Glycerol	-	-	-
	ERY	Erythritol	-	-	-
	DARA	D-arabinose	-	-	-
	LARA	L-arabinose	-	-	-
	RIB	D-ribose	-	-	-
	DXYL	D-xylose	-	-	-
	LXYL	L-xylose	-	-	-
	ADO	D-xylose	-	-	-
	MDX	Methyl-beta-D-xylopyranoside	-	-	-
	GAL	D-galactose	-	-	-
	GLU	D-glucose	+	+	+
	FRU	D-fructose	+	+	+
	MNE	D-mannose	-	-	-
	SBE	L-sorbose	-	-	-
	RHA	L-rhamnose	-	-	-
	DUL	Dulcitol	-	-	-
	INO	Inositol	-	-	-
	MAN	D-mannitol	-	-	-
	SOR	D-sorbitol	-	-	-
	MDM	Methyl-alpha-D-mannopyranoside	-	-	-
	MDG	Methyl-alpha-D-glucopyranoside	-	-	-
	NAG	N-acetylglucosamine	+	+	+
	AMY	Amygdalin	-	-	-
	ARB	Arbutin	+	+	+
	ESC	Esculin ferric citrate	+	+	+
	SAL	Salicin	+	+	+
	CEL	D-cellobiose	-	-	-
	MAL	D-maltose	+	+	+
	LAC	D-lactose (bovine origin)	-	-	-
	MEL	D-melibiose	-	-	-
	SAC	D-saccharose (sucrose)	+	+	+
	TRE	D-trehalose	+	+	+
	INU	Inulin	-	-	-
	MLZ	D-melezitose	-	-	-
	RAF	D-raffinose	-	-	-
	AMD	Amidon (starch)	+	+	+
	GLYG	Glycogen	+	+	+
	XLT	Xylitol	-	-	-
	GEN	Gentiobiose	-	-	-
	TUR	D-turanose	-	-	-

	LYX	D-lyxose	-	-	-
	TAG	D-tagatose	-	-	-
	DFUC	D-fucose	-	-	-
	LFUC	L-fucose	-	-	-
	DARL	D-arabitol	-	-	-
	LARL	L-arabitol	-	-	-
	GNT	Potassium gluconate	-	-	-
	2KG	Potassium 2-ketogluconate	-	-	-
	5KG	Potassium 5-ketogluconate	-	-	-
